# Optimized Treatment for Infantile Spasms: Vigabatrin versus Prednisolone versus Combination Therapy

**DOI:** 10.3390/jcm8101591

**Published:** 2019-10-02

**Authors:** Jongsung Hahn, Gyunam Park, Hoon-Chul Kang, Joon Soo Lee, Heung Dong Kim, Se Hee Kim, Min Jung Chang

**Affiliations:** 1 Department of Pharmacy and Yonsei Institute of Pharmaceutical Sciences, College of Pharmacy, Yonsei University, Incheon 21983, Korea; jshahn0220@gmail.com (J.H.); konamp@yonsei.ac.kr (G.P.); 2 Division of Pediatric Neurology, Department of Pediatrics, Epilepsy Research Institute, Severance Patients’s Hospital, Yonsei University College of Medicine, Seoul 03722, KoreaJOOSL96@yuhs.ac (J.S.L.); HDKIMMD@yuhs.ac (H.D.K.); 3 Department of Pharmaceutical Medicine and Regulatory Sciences, Colleges of Medicine and Pharmacy, Yonsei University, Incheon 21983, Korea

**Keywords:** Infantile spasms, First-line treatment, Combination of vigabatrin with prednisolone, Higher dose of prednisolone

## Abstract

Hormone therapies and vigabatrin are first-line agents in infantile spasms, but more than one-third of patients fail to respond to these treatments. This was a retrospective study of patients with infantile spasms who were treated between January 2005 and December 2017. We analyzed the response rates of initial treatment and second-line treatment. Responders were defined as those in whom cessation of spasms was observed for a period of at least one month, within 2 weeks of treatment initiation. Regarding the response rate to initial treatment, combination therapy of vigabatrin with prednisolone showed a significantly better response than that of vigabatrin monotherapy (55.3% vs. 39.1%, *p* = 0.037). Many drugs, such as clobazam, topiramate, and levetiracetam, were used as second-line agents after the failure of vigabatrin. Among these, no antiepileptic drug showed as good a response as prednisolone. For patients who used prednisolone, the proportion of responders was significantly higher in the higher-dose group (≥40 mg/day) than in the lower-dose group (66.7% vs. 12.5%, *p* = 0.028). Further studies of combination therapy to assess dosage protocols and long-term outcomes are needed.

## 1. Introduction

Infantile spasms is a rare and severe seizure disorder that is difficult to treat [1]. Its incidence ranges from 2 to 3.5 cases per 10,000 live births, with a peak onset at 3 to 7 months of age [2]. Infantile spasms possess three features: epileptic spasms, hypsarrhythmia on electroencephalogram (EEG), and developmental regression [3]. Delayed treatment can lead to poor outcomes; Therefore, timely and appropriate treatment is important [4].

Hormonal therapies (adrenocorticotropic hormone (ACTH) or prednisolone] and vigabatrin are the two main treatments that are considered “standard” for infantile spasms [5]. Hormonal treatment was first used in the treatment of this disorder in 1958, and has been shown to be effective in the cessation of spasms and resolution of EEG abnormalities [6]. Vigabatrin, an inhibitor of γ-aminobutyric acid transaminase, was approved by the US Food and Drug Administration for the treatment of infantile spasms in 2009 [7]. 

In the United Kingdom Infantile Spasms Study (UKISS) trial, hormonal therapies showed a higher rate of cessation of spasms at 2 weeks than vigabatrin (73% vs. 54%, *p *= 0·043) [8]. In addition, according to the 2012 American Academy of Neurology and Child Neurology Society evidence-based guideline, ACTH is more effective than vigabatrin, excluding patients with tuberous sclerosis complex [9]. However, some studies did not show significant differences in long-term efficacy between hormonal therapies and vigabatrin [10,11]. 

More than one-third of patients fail to respond, even if treated with standard therapies. In addition, all other antiepileptic drugs (e.g., valproic acid, pyridoxine, topiramate, zonisamide, and levetiracetam) have insufficient evidence for treating infantile spasms, when used as second-line therapy or add-on therapy [12,13,14,15,16,17]. Recently, there has been ongoing research on the additional efficacy of using vigabatrin and steroid combination treatment. Some studies have shown a short-term response rate that is significantly higher with combination treatment than with steroids alone [18,19], but such advantages are still unclear.

Therefore, we aimed to assess the efficacy of vigabatrin and steroid combination treatment in comparison with standard monotherapy as first-line treatments for infantile spasms. We also evaluated the response rates of various secondary medications in patients in whom these first-line treatments were ineffective.

## 2. Experimental Section

### 2.1. Study Design and Subjects

We conducted a retrospective analysis of the medical records of patients with infantile spasms who were treated and followed up for at least 3 months in a university-affiliated tertiary care hospital in Korea between January 2005 and December 2017. This study was approved by the Institutional Review Board (IRB No. 4-2016-0822) of Severance Hospital, and conducted in accordance with the principles of the STROBE statement which stands for an international, collaborative initiative of epidemiologists, methodologists, statisticians, researchers and journal editors involved in the conduct and dissemination of observational studies, with the common aim of STrengthening the Reporting of OBservational studies in Epidemiology. Written informed consent was waived by IRB because this study was based on retrospective medical record data, and all data were anonymized.

The inclusion criteria were as follows: a diagnosis of clinical spasms and EEG demonstrating features of hypsarrhythmia or modified hypsarrhythmia in patients between the ages of 2 and 12 months.

The exclusion criteria included the following: patients with uncertain diagnosis, patients with early infantile epileptic encephalopathy, patients who had concurrent treatment in other hospitals, patients with incomplete medical records, and patients with no follow-up visit.

### 2.2. Outcome Measurement

We considered vigabatrin and oral corticosteroids (all the cases in our analysis had been treated with prednisolone) as the standard therapies in this study. ACTH was excluded because of the lack of availability of this drug in Korea [20]. All other medications were considered non-standard therapies (e.g., clobazam, topiramate, valproic acid, levetiracetam, phenobarbital, zonisamide, clonazepam, oxcarbazepine, and a ketogenic diet). We analyzed the efficacy of the following four treatment groups: vigabatrin, prednisolone, combination therapy with vigabatrin and prednisolone, and non-standard therapy.

The primary clinical outcome was the response to initial treatment. Responders were defined as those whose clinical spasms were resolved within 2 weeks of treatment, and in whom this effect was sustained for at least 4 weeks [18,21]. Patients who did not have complete resolution of clinical spasms within 2 weeks of treatment or those in whom efficacy was sustained for less than 4 weeks were considered non-responders. In addition, we investigated the relapse rate of each treatment group. Clinical relapse was defined as the recurrence of clinical spasms within 6 months following a previous response to treatment.

As the secondary clinical outcome, we analyzed the responses to second-line treatment. Non-responders to the first-line treatment were the subjects of this analysis. The definitions of responder and non-responder are the same as those used for the primary clinical outcome. However, clinical relapse was not considered because the interval of follow-up for each patient was variable.

We collected the following data for each patient: sex, birthdate, report of EEG, presence of seizures prior to spasms, infantile spasm medication and dosage, age at spasm onset, first hospital visit date, age at initiation of treatment, treatment lag time (time from spasm onset to initiation of treatment), and time to the cessation of spasms.

### 2.3. Statistical Analysis

The demographic and clinical characteristics by treatment group were compared using Chi-square tests or Fisher’s exact test for categorical data, and Kruskal-Wallis tests for continuous data. The associations of clinical characteristics with treatment response were assessed using Chi-square tests or Fisher’s exact test. Next, the association between each treatment and response was assessed through multivariable logistic regression models that adjusted for factors found to be potentially predictive of response to therapy [22].

All statistical analyses were performed using SPSS**^®^** (IBM Corp. Released 2016. IBM SPSS Statistics for Windows, Version 24.0. Armonk, NY, USA: IBM Corp.), and two-sided tests with a significance level of α = 0.05 were used.

## 3. Results

### 3.1. Patient Characteristics

**Figure 1:**
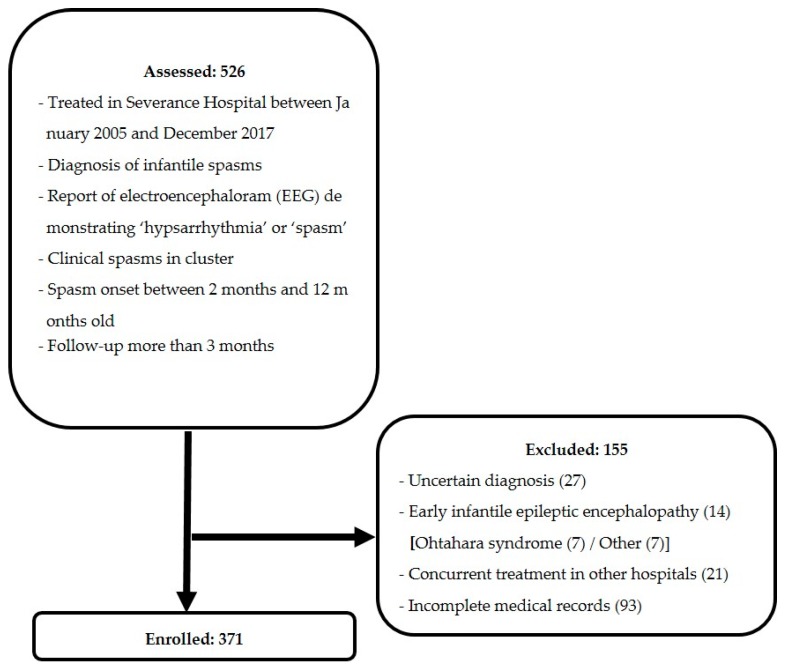
Flowchart of patient inclusion and exclusion criteria.

Figure 1 shows the flow chart of patients included in this study. Beginning with 526 patients, 155 patients were excluded, leaving 371 patients enrolled in our study.

### 3.2. First-Line Treatment

Among the 371 included patients, vigabatrin was used in 271 patients (73.0%), prednisolone was used in 6 patients (1.6%), combination therapy (vigabatrin and prednisolone) was used in 47 patients (12.7%), and non-standard therapy was used in 47 patients (12.7%). Seizures prior to spasms, age at spasm onset, age at treatment, and etiology were not different in the four treatment groups (Table 1). However, the treatment lag time was significantly different in each group, which may reflect prescription bias (12.0 (interquartile range (IQR), 5.0–29.0) vs. 7.0 (IQR 4.3–19.5) vs. 20.0 (IQR, 11.5–43.0) vs. 20.0 (IQR 7.0–56.0) days, *p* = 0.012, for the treatment groups, respectively).

Table 2 shows the response rates and periods from the start of treatment to response, and relapse rates of the first-line treatments. One hundred and forty-one of the 371 (38.0%) patients with infantile spasms responded to the initial treatment, with 106 out of 271 (39.1%) on vigabatrin, 4 out of 6 (66.7%) on prednisolone, 26 out of 47 (55.3%) on combination therapy, and 5 out of 47 (10.6%) on non-standard therapy. When comparing response rates between each treatment group, the vigabatrin group, prednisolone group, and combination group showed significantly better response rates than the non-standard group (*p* < 0.001, *p* = 0.005, and *p* < 0.001, respectively). We did not observe differences in the responses between the vigabatrin and prednisolone groups (*p* = 0.219) and between the combination and prednisolone groups (*p* = 0.687). However, the response rate of the combination group was significantly higher than that of the vigabatrin group (*p* = 0.037).

The cohorts of first-line treatment responders and non-responders were evaluated for predictors of outcome. There was no significant difference in response to first-line treatment when sex, age at spasm onset, and age at treatment were evaluated. However, treatment lag time and seizures prior to spasms were associated with response to therapy on univariate analysis (*p *< 0.001, and *p* = 0.030, respectively). In the multivariable logistic regression, the risk ratio of response to treatment decreased 0.406-times (95% confidence interval (CI) 0.239–0.689) in patients with lag times >4 weeks, compared with that in patients with lag times ≤4 weeks (*p* = 0.001). Patients with prior seizures were 0.593-times (95% CI 0.371–0.949) less likely to respond to therapy than patients without prior seizures (*p* = 0.029). Even after adjustment for treatment lag time and prior seizure history, comparisons of response rates from each treatment group showed the same results (Table 3).

### 3.3. Second-Line Treatment

Various drugs were used when vigabatrin, the most frequently used first-line treatment, failed. A high percentage of patients (128/165, 77.6%) received the following three drugs: clobazam, topiramate, or prednisolone. Specifically, among these 165 patients, 65 patients (39.4%) received clobazam, 43 patients (26.1%) received topiramate, and 20 patients (12.1%) received prednisolone. The remaining 37 patients (22.4%) received other antiepileptic drugs such as levetiracetam (11), zonisamide (8), phenobarbital (8), valproic acid (3), clonazepam (1), lamotrigine (1), or vigabatrin up-titration (5). Overall, 38 out of the 165 patients (23.0%) were observed to be spasm-free. Nine of the 20 patients (45.0%) receiving prednisolone, 12 of the 65 patients (18.5%) receiving clobazam, and 9 of the 43 patients (20.9%) receiving topiramate responded. Prednisolone showed a significantly higher response rate than both clobazam (*p* = 0.035) and topiramate (*p* = 0.049). There was no significant difference between the response rates of clobazam and topiramate (*p* = 0.751). The other patients who showed cessation of spasms (*n* = 8) were as follows: two out of 11 patients who received levetiracetam, 1 out of 8 patients who received zonisamide, and 5 patients who received up-titration of vigabatrin therapy. The responses to second-line therapy in all first-line treatment failure groups are presented in Table 4.

### 3.4. Dosage of Prednisolone

**Figure 2:**
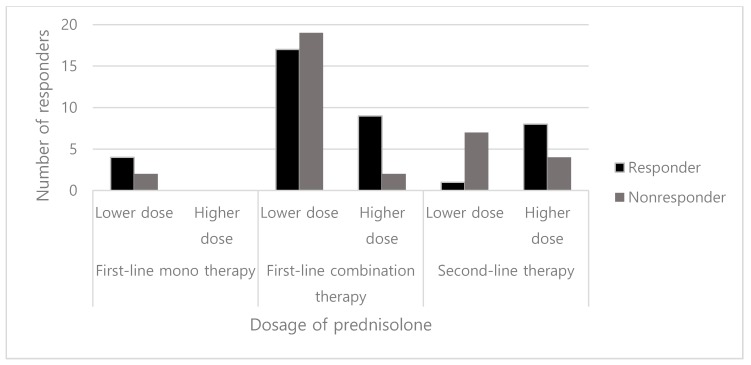
Number of responders by dosage of prednisolone.

We compared the efficacy of the higher dose (40–60 mg/day) of prednisolone with that of the lower dose (<40 mg/day). In the prednisolone monotherapy group, all patients were treated with 20 mg/day (lower dose), and 4 out of 6 of these patients responded. In the group of combination therapy with vigabatrin and prednisolone, 81.8% (9 out of 11) of the patients in the higher dose group responded compared to 47.2% (17 out of 36) of the patients in the lower dose group. However, this difference was not statistically significant (*p* = 0.081). When prednisolone was used as the second-line therapy after the failure of vigabatrin, the proportion of responders was significantly higher in the higher dose group than that in the lower dose group (8/12, 66.7% vs. 1/8, 12.5%, *p* = 0.028) (Figure 2).

## 4. Discussion

Optimized treatment for infantile spasms remains controversial. The results of this study suggest that an initial combination treatment of vigabatrin with prednisolone is more beneficial than vigabatrin monotherapy for patients with infantile spasm. After adjusting for treatment lag time and prior history of seizures, which were predictors of the response, the response rate with combination therapy was almost twice that of vigabatrin alone. This result could be interpreted in the same context as combination therapy yielding higher short-term response rates than hormonal treatment alone [18,19]. Combination therapy could have a synergistic effect, and prevent treatment delay by a rapid response [23]. Considering similar relapse rates and time to cessation of spasms between the vigabatrin and combination therapy groups in our study, we propose that combination therapy could be recommended at the start of treatment. However, further studies of combination therapy focusing on long-term efficacy, safety, factors influencing the response, and optimized dosing protocols (synchronous initiation or stepwise approach [24]) are needed.

In second-line treatments, which were used in cases of insufficient response to vigabatrin, prednisolone showed a greater response rate than non-standard treatments (45% vs. 17%, *p* = 0.008). In first-line prednisolone or combination treatment non-responders, additional vigabatrin (*n* = 2) or up-titration of vigabatrin (*n* = 1) showed 100% response rates, whereas additional non-standard treatment (*n* = 20) showed only a 10% response rate. These results were in agreement with a previous prospective study that showed standard therapy, which had a different mechanism of action from the initial treatment, was effective as a second-line treatment [13]. Although not as effective as standard treatments, non-standard therapies (i.e., clobazam, topiramate, and levetiracetam) elicited responses in some patients. After standard therapies have failed, these antiepileptic drugs can be considered as treatment options. However, to provide more evidence to justify this conclusion, studies with large patient cohorts are needed.

The response rate of prednisolone should be interpreted with caution because of the small sample size (first-line monotherapy, 6; first-line combination therapy, 47; second-line therapy, 21) and different dose protocols of prednisolone in our cohort of patients. Until 2015, our hospital patients tended to receive lower doses because of concerns regarding prednisolone’s serious adverse reactions, such as infection, sepsis, hypertension, adrenal insufficiency, growth retardation, and gastrointestinal bleeding [9]. However, Chellamuthu et al. reported that high-dose (4 mg/kg/day) prednisolone was more effective than standard dose (2 mg/kg/day) in spasm cessation at 14 days [25], and recent guidelines by the Pediatric Epilepsy Research Consortium (PERC) also recommended a prednisolone dosage of 10 mg four times daily [24]. When we analyzed response rates according to dosage of prednisolone, 12.5% of patients receiving low-dose prednisolone responded, whereas 66.7% receiving the high dose (≥40 mg/day) responded when used as second-line treatment after vigabatrin failure. Higher-dose prednisolone (up to 60 mg/day) has been shown to be safe in various studies [1,8,26]. Thus, we propose that the higher dose of prednisolone could be recommended for all patients with infantile spasms. Meanwhile, vigabatrin was administered at 50 mg/kg/day initially and increased to 100–150 mg/kg/day in most patients.

Our study has some limitations. First, as a nonrandomized, retrospective study, it was difficult to control the dosage and duration of treatment accurately, in a manner that could be done in a prospective study. However, our study does have an advantage of reflecting the clinical reality beyond randomized controlled trials. In clinical practice, drug efficacy was not assessed until after 2 weeks of use, and many patients were given additional drugs if the previous drug appeared ineffective. We assumed that the second treatment had caused a response even though the first treatment continued to be used during second-line treatment, and could have been responsible for the response observed. However, since two drugs were co-administered, the effect of each drug could not be measured accurately. In this context, we have been cautious in not overinterpreting our findings. Second, a response was defined as only the cessation of spasms without considering EEG resolution. According to a recent study, hypsarrhythmia is not seen in all patients with infantile spasms, and the presence or absence of hypsarrhythmia may not have predictive value [27]. Typically, clinical spasms are associated with developmental regression [28], which justifies our outcome measurement. Third, we did not identify distinct patient subsets that showed a better response to combination therapy than to vigabatrin alone. Further study considering each patient’s baseline characteristics, including etiology and developmental function, is needed.

Ultimately, this study had a larger number of patients for analysis than previous studies and provided evidence of effective treatments for managing infantile spasms. Although there were several limitations, it was meaningful that our study evaluated actual prescription patterns in clinical practice.

## Figures and Tables

**Table 1:** Baseline characteristics by type of first-line treatment.

Characteristic	Treatment Group ^a^					*p* Value
	Vigabatrin *n* = 271	Prednisolone *n* = 6	Vigabatrin + Prednisolone *n* = 47	Non-Standard *n* = 47	Total *n* = 371	
**Sex** **Male** **Female**	163 (60.1) 108 (39.9)	1 (16.7) 5 (83.3)	21 (44.7) 26 (55.3)	30 (63.8) 17 (36.2)	215 (58.0) 156 (42.0)	0.032 ^†,^*
**Presence of seizure history**	97 (35.8)	0 (0.0)	16 (34.0)	15 (31.9)	128 (34.5)	0.320 ^†^
**Age at spasm onset, mo.**	5.8 (4.4, 7.3)	7.7 (5.1, 8.7)	5.7 (4.4, 6.7)	5.7 (4.4, 7.4)	5.8 (4.4, 7.3)	0.719 ^‡^
**Age at treatment, mo.**	6.6 (5.3, 8.1)	8.1 (5.4, 8.9)	6.6 (5.5, 8.0)	7.1 (5.3, 8.8)	6.6 (5.3, 8.2)	0.804 ^‡^
**Lag time from spasm onset to treatment, days**	12.0 (5.0, 29.0)	7.0 (4.3, 19.5)	20.0 (11.5, 43.0)	20.0 (7.0, 56.0)	14.0 (5.0, 32.0)	0.012 ^‡,^*
**Etiology** **Structural** **Metabolic** **Infection** **Genetic** **Unknown**	87 (32.1) 23 (8.5) 6 (2.2) 7 (2.6) 148 (54.6)	1 (16.7) 0 (0) 0 (0) 0 (0) 5 (83.3)	15 (31.9) 2 (4.3) 0 (0) 1 (2.1) 29 (61.7)	12 (25.5) 2 (4.3) 1 (2.1) 1 (2.1) 31 (66.0)	115 (31.0) 27 (7.3) 7 (1.9) 9 (2.4) 213 (57.4)	0.938 ^§^

^a^ Values are n (%) or median (Q1, Q3); mo., months. ^†^ Chi-square test; ^‡^ Kruskal-Wallis test; ^§^ Fisher’s exact test. * *p*-value < 0.05.

**Table 2:** Effect of first-line treatment on response.

	Response to Initial treatment ^a^		Time to Cessation of Spasms (days) ^b^	Relapse Rate ^a^
Treatment	Response *n* = 141	Non-Response *n* = 230		
Vigabatrin	106 (39.1)	165 (60.9)	5 (3, 9)	8 (7.5)
Prednisolone	4 (66.7)	2 (33.3)	4 (3, 7)	1 (25.0)
Vigabatrin + prednisolone	26 (55.3)	21 (44.7)	5 (2, 9)	3 (11.5)
Non-standard	5 (10.6)	42 (89.4)	4 (2, 7)	2 (40.0)

^a^ Values are n (%); ^b^ Values are median (Q1, Q3).

**Table 3:** Relative probability (relative risk) of response to treatment.

Characteristic	Responders *n* (%)	*p *value ^†^	Crude Risk Ratio (95% CI)	Adjusted ^a^ Risk Ratio (95% CI)	Adjusted ^b^ Risk Ratio (95% CI)
**Treatment**					
Vigabatrin	106 (39.1)		REF	REF	REF
Prednisolone	4 (66.7)	0.219	3.113 (0.560, 17.296)	2.602 (0.465, 14.560)	2.980 (0.523, 16.975)
Vigabatrin + prednisolone	26 (55.3)	0.037 *	1.927 (1.032, 3.599)	1.929 (1.028, 3.620)	2.139 (1.124, 4.069)
Non-standard	5 (10.6)	<0.001 *	0.185 (0.071, 0.483)	0.179 (0.068, 0.468)	0.205 (0.078, 0.539)
**Seizure history**					
No	102 (42.0)	0.030 *	REF	REF	-
Yes	39 (30.5)		0.606 (0.385, 0.954)	0.593 (0.371, 0.949)	-
**Lag time**					
**≤**4 weeks	116 (43.8)	<0.001 *	REF	-	REF
>4 weeks	25 (23.6)		0.396 (0.238, 0.660)	-	0.406 (0.239, 0.689)

REF, reference group. ^†^ Chi-square test * *p*-value <0.05. ^a ^Model including treatment and seizures before infantile spasms as covariates. ^b ^Model including treatment and lag time as covariates.

**Table 4:** Response rate to second-line treatment.

	Second-Line Treatment	Vigabatrin	Prednisolone	Non-Standard
First-Line Treatment				
**Vigabatrin**		5/5, 100% (up-titration)	9/20, 45%	24/140, 17%
**Prednisolone**		2/2, 100%	-	-
**Vigabatrin + prednisolone**		1/1, 100% (up-titration)	-	2/20, 10%
**Non-standard**		13/31, 42%	0/1, 0%	2/10, 20%

## References

[1] Hancock E.C., Osborne J.P., Edwards S.W. (2013). Treatment of infantile spasms. Cochrane Database Syst. Rev..

[2] Pellock J.M., Hrachovy R., Shinnar S., Baram T.Z., Bettis D., Dlugos D.J., Gaillard W.D., Gibson P.A., Holmes G.L., Nordl D.R. (2010). Infantile spasms: A U.S. consensus report. Epilepsia.

[3] D’Alonzo R., Rigante D., Mencaroni E., Esposito S. (2018). West Syndrome: A Review and Guide for Paediatricians. Clin. Drug Investig..

[4] O’Callaghan F.J., Lux A.L., Darke K., Edwards S.W., Hancock E., Johnson A.L., Kennedy C.R., Newton R.W., Verity C.M., Osborne J.P. (2011). The effect of lead time to treatment and of age of onset on developmental outcome at 4 years in infantile spasms: Evidence from the United Kingdom Infantile Spasms Study. Epilepsia.

[5] Wilmshurst J.M., Gaillard W.D., Vinayan K.P., Tsuchida T.N., Plouin P., Van Bogaert P., Carrizosa J., Elia M., Craiu D., Jovic N.J. (2015). Summary of recommendations for the management of infantile seizures: Task Force Report for the ILAE Commission of Pediatrics. Epilepsia.

[6] Tsao C.Y. (2009). Current trends in the treatment of infantile spasms. Neuropsychiatr. Dis. Treat..

[7] Lerner J.T., Salamon N., Sankar R. (2010). Clinical profile of vigabatrin as monotherapy for treatment of infantile spasms. Neuropsychiatr. Dis. Treat..

[8] Lux A.L., Edwards S.W., Hancock E., Johnson A.L., Kennedy C.R., Newton R.W., O’Callaghan F.J.K., Verity C.M., Osborne J.P. (2004). The United Kingdom Infantile Spasms Study comparing vigabatrin with prednisolone or tetracosactide at 14 days: A multicentre, randomised controlled trial. Lancet.

[9] Go C.Y., Mackay M.T., Weiss S.K., Stephens D., Adams-Webber T., Ashwal S., Snead O.C. (2012). Evidence-based guideline update: Medical treatment of infantile spasms. Report of the Guideline Development Subcommittee of the American Academy of Neurology and the Practice Committee of the Child Neurology Society. Neurology.

[10] Lux A.L., Edwards S.W., Hancock E., Johnson A.L., Kennedy C.R., Newton R.W., O’Callaghan F.J.K., Verity C.M., Osborne J.P. (2005). The United Kingdom Infantile Spasms Study (UKISS) comparing hormone treatment with vigabatrin on developmental and epilepsy outcomes to age 14 months: A multicentre randomised trial. Lancet Neurol..

[11] Mohamed B.P., Scott R.C., Desai N., Gutta P., Patil S. (2011). Seizure outcome in infantile spasms—A retrospective study. Epilepsia.

[12] Hahn J., Lee H., Kang H.-C., Lee J.S., Kim H.D., Kim S.H., Chang M.J. (2019). Clobazam as an adjunctive treatment for infantile spasms. Epilepsy & Behavior.

[13] Knupp K.G., Leister E., Coryell J., Nickels K.C., Ryan N., Juarez-Colunga E., Gaillard W.D., Mytinger J.R., Berg A.T., Millichap J. (2016). Response to second treatment after initial failed treatment in a multicenter prospective infantile spasms cohort. Epilepsia.

[14] Rajaraman R.R., Lay J., Alayari A., Anderson K., Sankar R., Hussain S.A. (2016). Prevention of infantile spasms relapse: Zonisamide and topiramate provide no benefit. Epilepsia.

[15] Zhu X., Chen O., Zhang D., Jin R., Li F., Wang Y., Sun R. (2011). A prospective study on the treatment of infantile spasms with first-line topiramate followed by low-dose ACTH. Epilepsy Res..

[16] Lawlor K.M., Devlin A.M. (2005). Levetiracetam in the treatment of infantile spasms. Eur. J. Paediatr. Neurol..

[17] Suzuki Y., Nagai T., Ono J., Imai K., Otani K., Tagawa T., Abe J., Shiomi M., Okada S. (1997). Zonisamide monotherapy in newly diagnosed infantile spasms. Epilepsia.

[18] O’Callaghan F.J.K., Edwards S.W., Alber F.D., Hancock E., Johnson A.L., Kennedy C.R., Likeman M., Lux A.L., Mackay M., Mallick A.A. (2017). Safety and effectiveness of hormonal treatment versus hormonal treatment with vigabatrin for infantile spasms (ICISS): A randomised, multicentre, open-label trial. Lancet Neurol..

[19] Knupp K.G. (2017). Hormonal therapy with vigabatrin is superior to hormonal therapy alone in infantile spasms. J. Pediatrics.

[20] Sakakihara Y. (2011). Treatment of West syndrome. Brain Dev..

[21] Lux A.L., Osborne J.P. (2004). A proposal for case definitions and outcome measures in studies of infantile spasms and West syndrome: Consensus statement of the West Delphi group. Epilepsia.

[22] Kleinman L.C., Norton E.C. (2009). What’s the Risk? A simple approach for estimating adjusted risk measures from nonlinear models including logistic regression. Health Serv. Res..

[23] Hussain S.A. (2018). Treatment of infantile spasms. Epilepsia Open.

[24] Ko A., Youn S.E., Chung H.J., Kim S.H., Lee J.S., Kim H.D., Kang H.C. (2018). Vigabatrin and high-dose prednisolone therapy for patients with West syndrome. Epilepsy research.

[25] Chellamuthu P., Sharma S., Jain P., Kaushik J.S., Seth A., Aneja S. (2014). High dose (4 mg/kg/day) versus usual dose (2 mg/kg/day) oral prednisolone for treatment of infantile spasms: An open-label, randomized controlled trial. Epilepsy Res..

[26] Kossoff E.H., Hartman A.L., Rubenstein J.E., Vining E.P. (2009). High-dose oral prednisolone for infantile spasms: An effective and less expensive alternative to ACTH. Epilepsy Behav..

[27] Kelley S.A., Knupp K.G. (2018). Infantile Spasms-Have We Made Progress?. Curr. Neurol. Neurosci. Rep..

[28] Pavone P., Striano P., Falsaperla R., Pavone L., Ruggieri M. (2014). Infantile spasms syndrome, West syndrome and related phenotypes: What we know in 2013. Brain Dev..

